# Quantification of macular perfusion in healthy children using optical coherence tomography angiography

**DOI:** 10.1186/s40942-021-00328-2

**Published:** 2021-10-02

**Authors:** Fariba Ghassemi, Vahid Hatami, Farhad Salari, Fatemeh Bazvand, Hadi Shamouli, Masoumeh Mohebbi, Siamak Sabour

**Affiliations:** 1 Eye Research Center, Farabi Eye Hospital, Tehran University of Medical Sciences, Qazvin Square, 1336616351 Tehran, Iran; 2 Retina and Vitreous Service, Farabi Eye Hospital, Tehran University of Medical Sciences, Tehran, Iran; 3 Cornea Service, Farabi Eye Hospital, Tehran University of Medical Sciences, Tehran, Iran; 4 Department of Clinical Epidemiology, School of Health and Safety, Safety Promotion and Injury Prevention Research Centre, Tehran, Iran; 5 Department of Clinical Epidemiology, School of Health and Safety, Shahid Beheshti University of Medical Sciences, Tehran, Iran

**Keywords:** Choriocapillaris, Deep capillary plexus, Foveal avascular zone, Optical coherence tomography, Superficial capillary plexus, Vascular density

## Abstract

**Background:**

This study aimed at defining the variance of vessel density (VD) characteristics in the macula of children with normal eyes.

**Methods:**

This was a cross-sectional study in which subjects with normal eyes aged 3–18 years were enrolled. The macula was scanned by optical coherence tomography angiography (OCTA). Four age groups as under 7 years, 7–10 years, 11–14 years and more than 14 years of age were defined. The influences of age, gender, and body mass index on VD were analyzed.

**Results:**

A total of 108 normal eyes from 54 participants with a mean age of 10.9 years were enrolled in the study. At the superficial and deep retina capillary plexus (SCP and DCP) and choriocapillaris (CC), respectively, the mean VD of the fovea was 20.10%, 36.99%, and 75.67%; at parafovea, these measurements were 53.12%, 55.81%, and 69.76%; and at perifovea, these measures were 51.38%, 52.46%, and 73.47%. The median foveal avascular zone (FAZ) was 0.30 mm^2^. No significant differences between superior-hemi VD and inferior-hemi VD were found in the studied plexuses. The VD at parafovea and perifovea CC differed significantly between groups. There was no variation in VD of macular retina and CC between eyes or sex. FAZ areas were different between genders.

**Conclusions:**

No difference between eyes and genders in the retina and CC VD of macular area was noted. FAZ area was larger in male. Even though this is not a longitudinal study, it may provide us with hints about macular vascular development during puberty and clinical implications of OCTA in children.

## Introduction

Optical coherence tomography angiography (OCTA) is a relatively recent diagnostic modality that provides a rapid, noninvasive, high-resolution measurement of retinal and choroidal vascular layers and blood flow without the use of dye injection [1–3]. The ability to distinguish the retinal superficial (SCP) and deep (DCP) capillary plexus and analyze the various retinal vascular layers separately is a major advantage of OCTA over fluorescein angiography (FA) [4, 5].

The characteristics of vascular perfusion density in healthy children's eyes can be used to identify pathological OCTA findings associated with pediatric retinal vascular disorders such as Coats’ disease, hereditary exudative vitreoretinopathy, and others. Understanding the physiological changes that arise through infancy and adolescence, as well as gender discrepancies, is crucial in evaluating the pathophysiology of certain childhood retinal disorders. It is mysterious what physiological changes occur in the macula during the first decades of life. The aim of this research was to assess the structural and perfusion of macular region in observable 6 × 6 mm area at macular region. Despite its potential utility, vascular density (VD) data in normal children's eyes is limited, and clinical applications in children are also sparse [1, 6, 7]. There are few studies that have attempted to present normative data about children [6, 8, 9]. Thus, the objective of this research was to report macular microvascular measures in healthy Iranian pediatric individuals using OCTA analysis, as well as to assess the factors that may affect this quantitative data.

## Methods

Between April 2018 and May 2020, research was conducted on healthy children visiting Farabi Eye Hospital for routine eye exams, as well as on selected students from two Tehran schools. Institutional review board approval was obtained from Farabi Hospital Review Board-Tehran University of Medical Sciences (Tehran-Iran) for this cross-sectional study. It followed the tenets of the Declaration of Helsinki. A written informed consent was obtained from the participants. The parents of fifteen eligible children did not sign the informed consent form, so they were excluded. In our research, we were able to achieve a sufficient signal-to-noise ratio and experienced no loss of long fixation in the majority of the children due to the fixation target and verbal incentive. Despite several scans, 5 younger children (11%) refused to comply.

The study included consecutive healthy children with a best-corrected visual acuity (BCVA) of 20/20, refractive error between − 3 and + 1 diopter and a spherical equivalent (SE) of − 0.5 to + 0.5, and intraocular pressure (IOP) of less than 21 mmHg. Exclusion criteria were any condition precluding accurate retinal imaging, patient gesture, frequent blinking, and the history of prior migraine or any systemic, neurological, and ocular disease or syndromic condition, any media opacity, ophthalmic surgery or laser history, amblyopia, and any positive past medical, surgical and drug history and/or any trauma.

Patients were classified into four groups: those under the age of seven, those aged seven to ten, those aged eleven to fourteen, and those aged fourteen to eighteen. Full ophthalmologic examination including clinical history, past medical history, best-corrected visual acuity (BCVA, decimal scale), anterior segment biomicroscopy using a slit lamp, Goldmann applanation tonometry (or tonopen), and dilated pupil (1% tropicamide) ophthalmoscopy were performed.

Examinations and imaging were carried out on the same day in the morning working hours (8 up to 2 PM). OCTA images were obtained using the split-spectrum amplitude-decorrelation angiography algorithm (SSADA) on the AngioVue OCT-A system version 2018.0.0.18 (Optovue RTVue XR Avanti, California, USA). This device uses an 840 nm wavelength laser to capture 70,000 A-scans per second; 304 A-scans made up a B-scan, while 304 vertical (Y-FAST) and horizontal (X-FAST) lines were sampled in the scanning area to obtain a 3D data cube and eliminating any motion artifact.

Automated segmentation of SCP and DCP and choriocapillay (CC) was performed using the integral software algorithm which sets the inner margin of SCP at 3 μm below the internal limiting membrane (ILM) of the retina and the outer boundary at 15 μm beneath the inner plexiform layer (IPL), with the DCP at 15 μm beneath the IPL to 71 μm under the IPL. CC was determined as the area between 15 and 45 μm below the Bruch’s membrane.

VD was calculated by the software as the relative density of the flow (percentage) of the total studied area on the binary reconstructed images [10]. The fovea position is automatically determined by OCTA. The foveal region was outlined as a central circle with a diameter of 120 pixels (1.0 mm) and the parafoveal area was outlined as a 91-pixel wide ring around the foveal region (1.0 mm width).

For statistical analysis, the SCP, DCP and CC of foveal, parafoveal, perifoveal and whole image (WI) and superior (sup) and inferior (inf) half (hemi) data of parafoveal and perifoveal areas at macula on 6 × 6-mm volumetric scans, on the basis of the Early Treatment Diabetic Retinopathy Study (ETDRS) grid, were composed. The software automatically generated the VD, thickness, and area of the foveal avascular zone (FAZ) used in this analysis. FAZ area (in millimeters squared) measured in the 3 × 3-mm scan. Central macular thickness (CMT) was computed by an automated algorithm available in the machine.

Data from both eyes of all subjects was considered for analysis. Two ophthalmologists (FG and VH) performed the evaluation of the OCTA images. In OCTA-based quantitative tests, image quality is reported to be a possible confounder [8, 11]. As a result, all results in our study were adjusted for SSI, which was found to have a strong correlation with SCP, DCP, and CC density.

### Statistical analysis

All quantitative variables were reported as mean with standard deviation after evaluating the normality of distribution with the Kolmogorov- Smirnov test and histogram. The majority of continuous variables did not have a typical Gaussian distribution, and all were reported as the median with the range. All statistical analyses were performed by statistical software (SPSS software Version 21; SPSS, Inc., Chicago, IL, USA). Kruskal–Wallis test and one-way analysis of variance (ANOVA) were performed for nonparametric and parametric comparison, respectively. Accordingly, the Mann Whitney U test and post hoc analysis (Dunnett’s test) were used to compare the values between groups in nonparametric and parametric variables. In this study, collinearity for different variables was checked. P-values less than 0.05 were considered statistically significant.

## Results

A total of 108 eyes of 54 individuals were analyzed in this study. Mean age of all subjects was 10.9 ± 3.9 years (range: 3–18 years). Seventy two percent (78 eyes) of participants were male with a mean age of 10.7 ± 4.0 years, and 28.8% (30 eyes) were female with a mean age of 11.4 ± 3.6 years old. Mean weight was 39.2 ± 15.3 kg and mean height was 137.8 ± 21.7 cm. Among the four studied groups, 26 eyes (24.1%) were in less than 7 years age group, 26 eyes (24.1%) in the 7–10 years age group, 35 eyes (32.4%) in the 11–14 years group, and 21 eyes (19.4%) were in the older than 14 years age group (Table 1).

**Table 1:** Demographic characteristics of normal children in different age groups

Groups	Total	< 7	10-Jul	14-Nov	> 14	P-value
	(108 eyes)	(26 eyes)	(26 eyes)	(35 eyes)	(21 eyes)	
	(M ± SD)	(M ± SD)	(M ± SD)	(M ± SD)	(M ± SD)	
OD (%)	54 (50)	13 (24.1)	13 (24.1)	18 (33.3)	10 (18.5)	0.995
Sex-male (%)	78 (72.2)	20 (25.6)	20 (25.6)	25 (32.1)	13 (16.7)	0.637
BCVA (LogMAR)	0	0	0	0	0	–
Weight	39.25 ± 15.37	23.92 ± 5.23	31.61 ± 4.39	44.76 ± 6.82	62.00 ± 14.66	**< 0.001**
Height	137.80 ± 21.75	109.42 ± 10.38	133.38 ± 3.80	149.35 ± 10.22	163.38 ± 16.21	**< 0.001**

Bold values indicate the differences in the weight and height in the study groups

**BCVA best corrected visual acuity

### CMT

CMT was 252.34 ± 24.64 μm and being mildly thicker in the male in comparison with the female (253.27 ± 30.11 vs. 250.61 ± 28.82; P = 0.528, Pearson Chi-square). The mean CMT in each group was 250.15 ± 28.17, 247.27 ± 23.44, 250.88 ± 18.00 and 271.44 ± 44.92 μm in the aging groups, consequently (P = 0.004, ANOVA).

### VD

At the macular region, the median whole image (WI) VD of SCP, DCP, and CC was 50.66, 51.15, and 70.75 percent, respectively. In the foveal region, VD of DCP was significantly higher than that in SCP (36.99 ± 8.82 vs. 20.10 ± 7.32%, P < 0.001), comparable with in the parafoveal area (54.52 ± 7.39 vs. 51.66 ± 6.88%; P = 0.005) and perifovea area (51.70 ± 6.98 vs. 50.91 ± 4.61%; P = 0.078 borderline).

VD of SCP and DCP of the foveal, parafoveal and perifoveal area were similar in males and females and right and left eyes (P < 0.05), and no difference in superior and inferior hemifield was observed at parafoveal and perifoveal areas of macula (P > 0.05).

In a post hoc analysis of variance (ANOVA) test and nonparametric tests (Kruskal–Wallis test), in SCP and DCP, the WI and especially inf-hemi of WI showed slight decrease after age 14. In evaluation the subsegmental changes, more changes in the parafoveal and perifoveal VD at inferior half were shown after age 14 (Tables 2, 3).

**Table 2:** Vascular density in superficial capillary plexus (SCP) of normal children in different age groups

Groups VD/Reference group (median, %)	Total (108 eyes)	< 7 (26 eyes)	7–10 (26 eyes)	11–14 (35 eyes)	> 14 (21 eyes)	P-value Kruskal–Wallis test
WI < 7 7–10 11–14	50.66 (21.04–56.74)	51.83 (21.04–56.74)	51.76 (44.09–55.29) –*	51.17 (46.58–55.68) –* –*	49.53 (35.81–55.01) –* 0.035* 0.008*	0.085
Wi-sup-hemi < 7 7–10 11–14	51.01 (25.43–57.60)	51.67 (25.43–56.85)	50.77 (45.27–55.09) –*	50.99 (46.38–55.60) –* –*	49.68 (32.97–57.60) –* < 0.001* **0.049**	0.156
Wi-inf-hemi < 7 7–10 11–14	50.79 (12.29–56.63)	48.99 (12.29–56.63)	50.79 (43.01–55.49) –*	51.20 (39.01–56.37) –* –*	48.47 (39.01–54.03) –* 0.013* 0.002*	**0.034**
Fovea < 7 7–10 11–14	20.10 (± 7.32)	20.34 (± 8.29)	20.20 (± 7.20) –*	20.31 (± 6.20) –* –*	18.26 (± 8.02) –* –* –*	**0.863
Para-fovea < 7 7–10 11–14	53.12 (5.82–59.68)	51.34 (5.82–59.68)	51.36 (26.58–57.91) –*	53.88 (44.72–58.61) –* –*	51.14 (28.06–59.01) –* –* 0.014*	0.089
Para-sup- hemi < 7 7–10 11–14	53.79 (10.76–65.85)	51.83 (25.43–65.85)	50.77 (45.27–55.09) –*	50.99 (46.38–55.60) –* –*	49.68 (32.97–56.05) –* –* 0.047*	0.274
Para-inf- hemi < 7 7–10 11–14	52.79 (0.89–58.97)	50.77 (0.89–50.39)	53.68 (10.95–58.66) –*	53.15 (44.61–58.73) –* –*	50.41 (32.79–58.97) –* –* 0.005*	0.060
Perifovea < 7 7–10 11–14	51.38 (22.09–57.07)	52.17 (22.09–57.07)	51.56 (47.57–56.02) –*	51.54 (46.74–56.18) –* –*	56.19 (41.03–55.39) –* 0.024* 0.025*	0.171
Peri-sup- hemi < 7 7–10 11–14	51.72 (30.82–58.25)	52.43 (30.82–58.25)	51.73 (46.77–55.34) –*	51.38 (45.85–56.78) –* –*	50.43 (40.11–56.79) –* 0.056* –*	0.154
Peri-inf-hemi < 7 7–10 11–14	51.49 (13.32–57.33)	52.14 (13.32–56.54)	51.34 (47.34–56.70) –*	52.09 (46.33–57.33) –* –*	49.67 (40.58–53.99) –* 0.010* 0.011*	0.131
FAZ	0.28 (0.04–4.20)	0.27 (0.05–1.10)	0.28 (0.10–5.05)	0.29 (0.09–0.40)	0.28 (0.04–4.20)	0.228

In the bolded values, there are significant differences in four groups (Kruskal-Wallis test) and two by two analysis of the variables in the study groups (Mann Whitney U test and post hoc analysis (Dunnett's test))

*Mann–Whitney test

**Kruskal–Wallis Test

**Table 3:** Vascular density in deep capillary plexus (DCP) of normal children in different age groups

Groups VD/Reference group (median, %)	Total (108 eyes)	< 7 (26 eyes)	7–10 (26 eyes)	11–14 (35 eyes)	> 14 (21 eyes)	P-value Kruskal–Wallis test
WI < 7 7–10 11–14	51.15 (16.36–63.32)	50.23 (16.36–63.32)	51.41 (43.53–61.12) –*	52.93 (42.62–61.51) –* –*	49.14 (28.51–58.65) –* 0.054* 0.018*	0.300
WI-sup-hemi < 7 7–10 11–14	50.91 (22.81–62.88)	50.66 (23.50–62.88)	51.07 (42.97–61.87) –*	52.29 (42.58–61.36) –* –*	50.44 (22.81–60.14) –* –* 0.028*	0.379
WI-inf-hemi < 7 7–10 11–14	51.38 (8.93–63.32)	50.33 (8.93–63.32)	51.83 (42.22–60.40) –*	52.70 (42.66–61.65) –* –*	50.04 (39.95–57.20) –* 0.036* 0.018*	0.186
Fovea < 7 7–10 11–14	36.99 (± 8.82)	35.86 (± 10.73)	38.12 (± 10.81) –*	37.79 (± 6.51) –* –*	34.84 (± 6.39) –* –* –*	**0.290
Para-fovea < 7 7–10 11–14	55.81 (6.75–64.83)	55.97 (6.75–64.83)	54.62 (33.28–62.90) –*	56.34 (48.67–63.47) –* –*	51.14 (28.06–59.01) –* –* 0.035*	0.404
Para-sup-hemi < 7 7–10 11–14	54.72 (7.01)	54.29 (9.66)	54.98 (5.20) –*	56.00 (4.21) –* –*	52.49 (9.10) –* –* –*	**0.519
Para-inf-hemi < 7 7–10 11–14	55.35 (0.89–58.97)	50.77 (0.89–50.39)	53.68 (10.95–58.66) –*	53.15 (44.61–58.73) –* –*	50.41 (32.79–58.97) –* –* 0.027*	0.420
Perifovea < 7 7–10 11–14	52.46 (16.88–65.79)	51.37 (16.88–65.79)	52.93 (44.34–61.67) –*	53.01 (44.56–62.58) –* –*	50.62 (31.76–59.31) –* 0.028* 0.007*	0.169
Peri-sup-hemi < 7 7–10 11–14	51.64 (23.81–67.10)	51.42 (23.86–67.10)	52.29 (42.51–61.80) –*	52.11 (44.10–61.95) –* –*	50.61 (23.81–60.82) –* –* 0.026*	0.336
Peri-inf-hemi < 7 7–10 11–14	51.73 (7.20)	49.82 (11.10)	53.35 (4.29) –*	53.50 (4.75) –* –*	49.45 (6.19) –* 0.008* 0.004*	0.060

In the bolded values, there are significant differences in four groups (Kruskal-Wallis test) and two by two analysis of the variables in the study groups (Mann Whitney U test and post hoc analysis (Dunnett's test))

*Mann–Whitney test

**Kruskal–Wallis Test

The most compacted VD of macular area was found at CC especially in the foveal area. At CC, the foveal VD decreased continually after age 11 (Mann–Whitney Test- Table 4). The VD of CC increases more in the perifoveal area between 7 and 14 years of age, after which it tends to decrease slightly. VD of CC increased in the parafoveal area after age 11 and then decreased after age 14 in the parafoveal area. Age was not associated with SCP, DCP, or CC once we took gender and BMI into account.

**Table 4:** Choriocapillaris (CC) vascular density of normal children in different age groups

Groups VD/Reference group (median, %)	Total (108 eyes)	< 7 (26 eyes)	7–10 (26 eyes)	11–14 (35 eyes)	> 14 (21 eyes)	P-value Kruskal–Wallis test
WI < 7 7–10 11–14	70.75 (22.76–78.90)	69.00 (22.76–74.80)	73.40 (63.68–77.41) 0.019*	73.43 (67.26–78.90) 0.001* –*	72.89 (62.68–76.76) –* –* 0.012*	**0.001**
WI-sup-hemi < 7 7–10 11–14	73.01 (40.48–79.62)	70.07 (40.48–76.05)	73.44 (64.22–77.80) < 0.17*	74.20 (66.73–79.62) < 0.001* –*	73.29 (63.72–78.25) –* –* 0.019*	**0.001**
WI-inf-hemi < 7 7–10 11–14	72.40 (0.92–78.36)	68.28 (0.92–74.91)	73.72 (62.78–77.36) 0.027*	73.03 (64.82–78.20) 0.003* –*	72.72 (56.39–75.36) –* –* 0.010*	**0.001**
Fovea < 7 7–10 11–14	75.67 (21.48–83.42)	76.02 (21.48–80.80)	77.43 (39.89–83.42) –*	75.49 (66.76–80.23) –* –*	73.95 (31.93–78.89) –* 0.014* 0.004*	**0.417
Para-fovea < 7 7–10 11–14	69.76 (9.61–80.95)	68.18 (9.61–76.43)	70.46 (57.64–80.22) –*	72.69 (61.20–80.95) 0.017* –*	69.43 (58.49–78.39) –* –* 0.005*	**0.013**
Para-sup-hemi < 7 7–10 11–14	70.23 (18.11–80–51)	69.50 (18.11–76.25)	69.99 (58.47–79.53) –*	72.76 (60.24–80.51) 0.037* –*	69.18 (56.53–77.59) –* –* 0.019*	**0.042**
Para-inf-hemi < 7 7–10 11–14	70.21 (1.10–81.38)	67.50 (1.10–76.61)	70.73 (46.22–80.90) –*	71.87 (62.16–81.38) 0.011* –*	69.50 (51.96–79.19) –* –* 0.007*	**0.013**
Perifovea < 7 7–10 11–14	73.47 (22.92–80.03)	69.38 (22.92–75.31)	73.76 (63.22–77.87) 0.017*	74.07 (67.06–80.03) 0.001* –*	73.72 (63.63–78.11) –* –* 0.048*	**0.001**
Peri-sup-hemi < 7 7–10 11–14	73.13 (44.91–78.94)	70.88 (44.91–76.61)	73.42 (63.97–77.93) 0.012*	74.79 (44.10–61.95) < 0.001* –*	73.77 (65.70–78.94) –* –* 0.027*	**0.001**
Peri-inf-hemi < 7 7–10 11–14	73.28 (0.81–79.04)	68.86 (0.81–75.66)	74.29 (62.47–77.80) < 0.025*	74.10 (65.33–79.04) 0.003* –*	74.12 (57.65–77.29) –* –* 0.048*	**0.002**

In the bolded values, there are significant differences in four groups (Kruskal-Wallis test) and two by two analysis of the variables in the study groups (Mann Whitney U test and post hoc analysis (Dunnett's test))

*Mann–Whitney test

**Kruskal–Wallis Test

### FAZ

In all participants the mean FAZ was 0.44 ± 0.78 mm^2^ (median = 0.30, 0.02–5.05); being significantly higher in the female in comparison with the male (median: 0.37 (0.05–1.08) vs. 0.28 (0.02–5.05), P = 0.013). The analysis showed no change in FAZ in the aging groups (P = 0.228, Mann Whitney) (Table 2). No inter-eye correlation was observed in the FAZ (P = 0.719, Spearman correlation). The results showed that there is no significant correlation between age and BMI with FAZ area (P > 0.05). There was no statistically significant correlation between FAZ and CMT (P = 0.274).

### Correlations

In our sample, there was no significant association between right and left eye for any of the study parameters, as predicted (P > 0.05). In addition, we performed the entire analysis considering one eye per subject, and the results were similar. For example, OCTA parameters’ correlation coefficients for bilateral eyes were less than 0.3 for FAZ (r = 0.25, P = 0.719), VD of foveal SCP (r = − 0.07, P = 0.84), foveal DCP (r = − 0.22, P = 0.619) and foveal CC (r = − 0.04, P = 0.669).

Correlations were evaluated after adjusting for the age, gender and Avg-SSI, and BMI and taking collinearity into account in multiple linear regression in these pediatric groups. The following results have been obtained:

*CMT* There was no correlation between CMT and any other parameter.

*SCP-fovea* was significantly correlated with DCP-fovea (B = 0.839, CI: 0.592–0.838, P < 0.001).

*DCP-fovea* was related with FAZ (B = − 0.181, CI: − 3.980 to − 0.918, P = 0.002), CMT (B = 0.105, CI: 0.001–0.066, P = 0.046), SCP-fovea (B = 0.634, CI: 0.612–0.876, P < 0.001), DCP-perifovea (B = 0.137, CI: 0.019–0.376, P = 0.030), WI-CC (B = 6.835, CI: 1.945–28.161, P = 0.025), CC-WI-sup-hemi (B = − 3.306, CI: − 13.964 to − 0.929, P = 0.026), and CC-WI-inf-hemi (B = − 3.958, CI: − 14.633 to − 0.914, P = 0.027).

*CC fovea* was associated with age (B = − 0.261, CI: 0.946 to − 0.349, P < 0.001), DCP-fovea (B = − 0.133, CI: 0.009–0.267, P = 0.036), SCP-parafovea (B = − 0.181, CI: − 3.980 to − 0.918, P = 0.008), CC-WI-inf-hemi (B = 2.743, CI: 1.033–4.710, P = 0.001), and CC-parafovea (B = 0.395, CI: 0.002–0.939, P = 0.049).

*FAZ* was correlated with SCP-fovea (B = -0.424, CI: − 0.052 to − 0.022, P < 0.001), CC-WI (B = − 1.841, CI: − 0.486 to − 0.115, P = 0.002), and CC-perifovea (B = 1.727, CI: 0.100–0.460, P = 0.003).

## Discussion

In groups of healthy pediatric subjects, we collected quantitative data on macular microvascular structure changes. FAZ and VD of SCP and DCP are unaffected by age or contralaterality. FAZ was greater in girls and could be impacted adversely by SCP-fovea and CC perfusion. DCP-fovea was found to be influenced by SCP-fovea (and vice versa), SCP-perifovea, FAZ, and VD of CC. The fovea has the highest mean of VD at CC, and the age groups have more VD in the inferior hemifield of CC at the macular region. The only variables that were correlated with VD of foveal CC were WI-inf-hemi-CC and SCP-parafovea.

Retinal and choroidal vascular changes may occur before structural changes. Many aspects of the microscopic and physiologic characteristics of the retina and choroid are now well understood in normal subjects thanks to OCTA. OCTA was used to explore pathophysiologic features of certain diseases [12–16]. Most previous studies using OCTA, however, mainly have evaluated normal adult patients [1, 4, 5, 9].

FAZ was reported to be 0.26–0.47 mm using different kinds of OCTA machines at 3 × 3-mm enface AngioScans in the kids between mean age of 8–12 years in different capillary layers. Hsu et al. recorded an FAZ of 0.35 ± 0.17 mm^2^ in their 89 eyes (mean age, 8.5 ± 5.3 years-Heidelberg OCTA instrument and MATLAB program by unknow size of evaluated field) [7]. Comparingly, Yilmaz et al. reported the SCP-FAZ area to be 0.28 ± 0.09 mm^2^ and DCP-FAZ to be 0.38 ± 0.09 in 15 eyes from 15 normal children (a mean age of 8.6 ± 2.2 years- Nidek’s RS-3000 in 3 × 3-mm) [17]. İçel et al. stated SCP-FAZ as 0.3 ± 0.09 mm^2^ in 146 children eyes (the mean age of 11.27 ± 3 years- Nidek RS-3000 AngioScan in 3 × 3 mm) [18]. In our study, the mean FAZ area was 0.44 ± 0.78 mm^2^, considering a single layer measurement on 3 × 3 mm enface image (automatedly derived data from software).

The observed differences may be attributed to ethnic differences, sample size, different OCTA machine used, and different capillary layer FAZ measurements. The FAZ area of adults has been found to be from 0.24 to 0.46 mm and more in DCP than SCP [4, 19–21]. Despite the different measurement techniques, the FAZ areas for healthy adults and children are comparable.

The effect of aging on the size and shape of the FAZ is a topic of hot debates. OCTA has demonstrated in many studies that the scale and form of the FAZ in healthy adult populations increases with age [6, 22, 23]. Since our and other studies only included children, the aging impact on FAZ could not be detected in this age range [7, 19, 24].

The impact of sex on the FAZ area remains inconclusive. Some research results indicated that adult females had higher FAZ than males, which can also be seen in children as in our study [8, 19, 23, 25, 26]. Other studies reported no significant difference between the sexes [18, 25, 26]. In our study, accounting our smaller number of female subjects, the median of FAZ was 0.28 mm (range 0.02–5.05) in male and 0.37 mm (range 0.05–1.08) in female. Population-based studies are needed to determine the true effect of sex on FAZ area in the future.

Unlike previous studies [6–8, 18, 26], our research found no correlation between FAZ and CMT. The difference may be attributed to unadjusted results to certain major confounders in previous studies.

The multivariate data analysis only revealed a correlation between FAZ and SCP, CC, especially perifoveal CC. For every 0.4 percent decrease in SCP-fovea and every 1.81 percent decrease in CC-WI VD, FAZ increased by 1 mm. Zhang et al. disclosed the impact of sex, foveal retinal thickness, parafoveal retinal thickness, axial length, and SSI on FAZ in their cohort [8].

Using the Optovue Angioretina program, several researchers have tested the SCP and DCP in healthy adult subjects. In line with our results, some of these studies revealed no variations in VD between genders [7] whereas increased SCP-VD in males was reported in other studies [6].

In our study, the foveal region CC had the most VD compared with the other parts in SCP, DCP, and CC. In the foveal region of CC, VD increased up to the age of 10 and then decreased from 11 to 18 years old. The parafoveal area of CC and mostly perifoveal area had increasing order up to 13 years of age and then mild decrease in each part was noted (P < 0.05). Similar to our results, Zhang et al. showed that in the parafoveal region, the CC had higher vascular flow compared to DCP and SCP (*P* < 0.001) [8]. Some studies have shown that as the FAZ area increases, the VD of SCP, DCP, and CMT decreases dramatically during aging [18]. In a pediatric study, it is found that CC perfusion density, not retinal VD inversely is correlated with age, even after accounting for potentially confounding factors such as refractive error and race, but not adjusting for sex and the BMI of the children [6]. Our study used 6 × 6-mm enface images for measurements to evaluate the macular region as a whole, while their study used 3 × 3-mm enface images, so the quantities are not equivalent.

Iafe et al. [22] analyzed adult macular VD in 70 subjects (mean 48 ± 20 years of age, range 9–88 years), Wang et al. [27] in 105 Chinese healthy participants (age: 35.9 ± 13.8 years, range 17–64 years), Coscas et al. [20] in 70 subjects (age: 48 ± 18 years, range 20–79 years), and Ghassemi et al. [5] in 112 healthy cases (age: 36.39 ± 11.31 years, range 12–67 years). VD of SCP and DCP decreased with age in these and other similar studies on healthy adult eyes [28, 29]. However, only the CC-fovea was affected by age in our pediatric series, and aging changes could be observed in children aged 11–18 years. We studied pediatric patients; therefore, we could not determine whether VD continues to decline through young adulthood or stabilizes.

Although we did not note any gender differences in VD of retina and CC at macular area, some significant differences were reported in the SCP between sexes in healthy children in other study [25]. On the other hand in adults, Coscas et al. uncovered a higher retinal VD in females than in males over-60 year-olds of age and attributed this difference to a slower vascular aging in females [20]. Other studies stated no gender-based variance in VD of retina in macular area [5].

The primary limitation of our study was the cross-sectional nature of the analysis and relatively small samples in age groups. Furthermore, we did not perform axial length measurements in our study that may have weakened our analysis. Although, we limited our cases to those with a small amount of refractive error and a spherical equivalent between − 0.5 and + 0.5 diopter, in which it does not seem to affect significantly the image magnification. The effect of ethnic variation on VD is not evaluated in this study. The flow projection artifact of the large vessels of the SCP onto deeper retinal layers may have been a concern and may have influenced the quantitative analysis of VD values in the DCP.

## Conclusion

Using OCTA as a noninvasive and accurate method, we created a valid normative database of choroidal and retinal microvasculature in healthy Iranian children. VD is similar in both sexes and eyes in this study data analysis and foveal VD was shown to be stable over time in children and not correlated with BMI. Interestingly, only the CC was affected by age in our pediatric series, and it tend to be decreased at 14–18 years. This database could be useful to evaluate foveal and choroidal development and pathophysiologic characteristics. Further research, including longitudinal follow-ups of children using hand-held SD-OCTA devices, would provide more information about the pattern in VD of retina and CC and FAZ during normal childhood development. Computing the VD is a feasible value for quantitative evaluation of macular perfusion status with potential clinical applications.

## Data Availability

The data that support the findings of this study are available from the authors upon reasonable request and after permission of Farabi Eye Hospital managing group and research center.

## Abbreviations

BMI: Body mass index
CC: Choriocapillaris
CMT: Central macular thickness
DCP: Deep capillary plexus
FAZ: Foveal avascular zone
OCT: Optical coherence tomography
SCP: Superficial capillary plexus
VD: Vascular density
WI: Whole image
